# Deficiency in the DNA repair protein ERCC1 triggers a link between senescence and apoptosis in human fibroblasts and mouse skin

**DOI:** 10.1111/acel.13072

**Published:** 2019-11-18

**Authors:** Dong Eun Kim, Martijn E. T. Dollé, Wilbert P. Vermeij, Akos Gyenis, Katharina Vogel, Jan H. J. Hoeijmakers, Christopher D. Wiley, Albert R. Davalos, Paul Hasty, Pierre‐Yves Desprez, Judith Campisi

**Affiliations:** ^1^ Buck Institute for Research on Aging Novato CA USA; ^2^ Centre for Health Protection Research National Institute of Public Health and the Environment (RIVM) Bilthoven The Netherlands; ^3^ Department of Molecular Genetics Erasmus University Medical Center Rotterdam The Netherlands; ^4^ Princess Máxima Center for Pediatric Oncology ONCODE Institute Utrecht The Netherlands; ^5^ CECAD Forschungszentrum Köln Germany; ^6^ Department of Molecular Medicine Sam and Ann Barshop Institute for Longevity and Aging Studies University of Texas Health Science Center San Antonio TX USA; ^7^ Lawrence Berkeley National Laboratory Berkeley CA USA

**Keywords:** aging, cell death, DNA damage repair, senescence‐associated secretory phenotype, tumor necrosis factor α

## Abstract

ERCC1 (excision repair cross complementing‐group 1) is a mammalian endonuclease that incises the damaged strand of DNA during nucleotide excision repair and interstrand cross‐link repair. *Ercc1^−/Δ^* mice, carrying one null and one hypomorphic *Ercc1* allele, have been widely used to study aging due to accelerated aging phenotypes in numerous organs and their shortened lifespan. *Ercc1^−/Δ^* mice display combined features of human progeroid and cancer‐prone syndromes. Although several studies report cellular senescence and apoptosis associated with the premature aging of *Ercc1^−/Δ^* mice, the link between these two processes and their physiological relevance in the phenotypes of *Ercc1^−/Δ^* mice are incompletely understood. Here, we show that ERCC1 depletion, both in cultured human fibroblasts and the skin of *Ercc1^−/Δ^* mice, initially induces cellular senescence and, importantly, increased expression of several SASP (senescence‐associated secretory phenotype) factors. Cellular senescence induced by ERCC1 deficiency was dependent on activity of the p53 tumor‐suppressor protein. In turn, TNFα secreted by senescent cells induced apoptosis, not only in neighboring ERCC1‐deficient nonsenescent cells, but also cell autonomously in the senescent cells themselves. In addition, expression of the stem cell markers p63 and Lgr6 was significantly decreased in *Ercc1^−/Δ^* mouse skin, where the apoptotic cells are localized, compared to age‐matched wild‐type skin, possibly due to the apoptosis of stem cells. These data suggest that ERCC1‐depleted cells become susceptible to apoptosis via TNFα secreted from neighboring senescent cells. We speculate that parts of the premature aging phenotypes and shortened health‐ or lifespan may be due to stem cell depletion through apoptosis promoted by senescent cells.

## Graphical abstract

ERCC1‐depleted cells become susceptible to apoptosis via TNFalpha secreted from neighboring senescent cells. Part of the premature aging phenotypes in ERCC1‐deficient mice may be due to stem cell depletion through apoptosis promoted by cellular senescence.

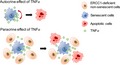

## 1 INTRODUCTION

The accumulation of DNA damage is thought to be a major driver of age‐related degeneration and pathologies and is considered one of the hallmarks of aging (Hasty, Campisi, Hoeijmakers, van Steeg, & Vijg, 2003). The major defenses against the genotoxic and cytotoxic consequences of DNA damage are two cell fates: cellular senescence and apoptosis. To prevent the propagation of damaged or dysfunctional cells, senescence arrests proliferation, essentially irreversibly, but senescent cells remain viable and metabolically active. By contrast, apoptosis is a form of programmed cell death and therefore effectively eliminates damaged or dysfunctional cells. Despite being distinct cell fates with different outcomes, both can be beneficial or deleterious, depending on the physiological context and age (Campisi, 2013; Childs, Baker, Kirkland, Campisi, & van Deursen, 2014). A major benefit of both senescence and apoptosis is the ability to suppress the development of cancer. However, both cell fates can also eventually compromise the ability of tissues to regenerate and/or function optimally.

Senescent cells accumulate during aging and at sites of many age‐related pathologies (Campisi, 2013; Coppe, Desprez, Krtolica, & Campisi, 2010). The persistent presence of senescent cells can foster chronic inflammation, modify tissue microenvironments, and alter the functions of neighboring cells—all owing to the secretion of numerous factors, including pro‐inflammatory cytokines, chemokines, growth factors, and proteases, termed the senescence‐associated secretory phenotype (SASP) (Campisi, 2013; Coppe et al., 2010, 2008; Davalos et al., 2013; Kuilman, Michaloglou, Mooi, & Peeper, 2010). Using transgenic mice in which senescent cells can be selectively eliminated, both lifelong and late‐life clearance were shown to extend median lifespan and attenuate several deleterious age‐associated phenotypes; these benefits were apparent in both progeric and naturally aged mice (Baker et al., 2011). Thus, cellular senescence has emerged as an important causative driver of many aging phenotypes and pathologies. Likewise, many aging phenotypes and pathologies can arise owing to deficits in the control of apoptosis. Resistance to apoptosis due to acquired mutations can promote cancer, whereas overactivation of apoptosis can cause tissue atrophy (Childs et al., 2014).

Cellular senescence and apoptosis share certain characteristics, including the ability to suppress tumorigenesis and potential to contribute to aging and age‐associated diseases. They also share certain regulatory axes, such as control by the p53‐p21 and PTEN‐PI3K‐Akt pathways (Childs et al., 2014). Depending on the degree of stress and type of DNA damage, as well as the cell type, state, and history, cells may undergo either senescence or apoptosis. Interestingly, senescent cells are generally resistant to apoptosis (Childs et al., 2014; Yosef et al., 2016). Nonetheless, senescent endothelial cells can become susceptible to apoptosis upon reduced BCL‐2 or increased BAX expression (Zhang, Patel, & Block, 2002) or reduced nitric oxide synthase (eNOS) (Li, Guo, Dressman, Asmis, & Smart, 2005; Matsushita et al., 2001). The molecular mechanisms linking senescence and apoptosis, especially driven by DNA damage, have not been extensively explored.

ERCC1 (excision repair cross complementing‐group 1) is a DNA repair protein that forms a heterodimer with XPF (xeroderma pigmentosum group F‐complementing protein, also known as ERCC4) and functions as a 5′‐3′ structure‐specific endonuclease. It participates in a number of DNA repair pathways in mammalian cells (Dolle et al., 2011; Weeda et al., 1997). The heterodimer is required for incising the damaged strand of DNA during nucleotide excision repair (NER) and the mechanistically related transcription‐coupled repair (TCR), and for resolving DNA interstrand cross‐links by interstrand cross‐link repair. In addition, the ERCC1/XPF endonuclease is important for single‐strand annealing of persistent double strand breaks (Marteijn, Lans, Vermeulen, & Hoeijmakers, 2014).

Because ERCC1/XPF participates in multiple DNA repair pathways, ERCC1/XPF mutations in humans are associated with several distinct syndromes. These syndromes include Cockayne and cerebro‐oculo‐facial‐skeletal (COFS) syndromes characterized by severe progressive developmental deficiencies and neurological abnormalities, as well as many other aging phenotypes and an extremely short lifespan (Gregg, Robinson, & Niedernhofer, 2011; Jaspers et al., 2007; Niedernhofer et al., 2006). They also include the cancer‐prone disorders xeroderma pigmentosum, Fanconi anemia, and XFE syndrome (Marteijn et al., 2014). An important tool for studying these diverse syndromes is *Ercc1^−/Δ^* mice. These mice lack one functional *Ercc1* allele and are hemizygous for a single truncated allele encoding a hypomorphic Ercc1 variant that lacks the last seven amino acids (Dolle et al., 2011; Weeda et al., 1997). The lifespan of this mouse is significantly truncated (4–6 months) and the animals show numerous premature aging phenotypes, including decreased body weight, prominent global neurodegeneration, and bone marrow failure and atrophy; they also show age‐associated pathology in major organs, such as the liver, kidney, skeletal muscles, and vasculature, although in their short lifespan they do not develop overt neoplastic lesions (Vermeij, Hoeijmakers, & Pothof, 2016). Several groups have described the presence of senescent cells in *Ercc1^−/Δ^* mice and suggested a role for these cells in accelerating aging phenotypes and pathologies when there is a defect in DNA damage repair (Robinson et al., 2018; Tilstra et al., 2012; Weeda et al., 1997). Concomitantly, apoptosis and its link to tissue atrophy and pathologies in the *Ercc1^−/Δ^* mice have been observed by other groups (Niedernhofer et al., 2006; Takayama et al., 2014). It is unclear whether and how these two distinct cell fates are linked in this DNA damage‐driven, premature aging mouse model.

Here, we show that DNA damage driven by deficient ERCC1 expression or activity promotes cellular senescence in human cells in culture and mouse skin *in vivo*. Senescence induced by ERCC1 downregulation depended on p53 expression, which earlier work showed restrains the SASP (Coppe et al., 2008; Rodier et al., 2009). We also found that ERCC1‐deficient cells undergo apoptosis, driven primarily by the SASP factor TNFα. TNFα induced apoptosis via extrinsic apoptotic signaling pathways, and TNFα secretion from ERCC1‐depleted senescent cells promoted apoptosis in neighboring nonsenescent cells as well as senescent cells themselves. We confirmed an increase in apoptotic cells in the skin of *Ercc1^−/Δ^* mice during aging, which was not detected in skin samples from age‐matched wild‐type littermates. We also found substantial depletion of epithelial stem cells, possibly due to apoptosis, in older *Ercc1^−/Δ^* mouse skin. Finally, we determined that the SASP factor TNFα accelerated apoptosis in ERCC1‐depleted cells, which likely contributes to the premature aging phenotypes and tissue dysfunction in *Ercc1^−/Δ^* mice.

## 2 RESULTS

### 2.1 ERCC1 deficiency promotes cellular senescence in skin

**Figure 1:**
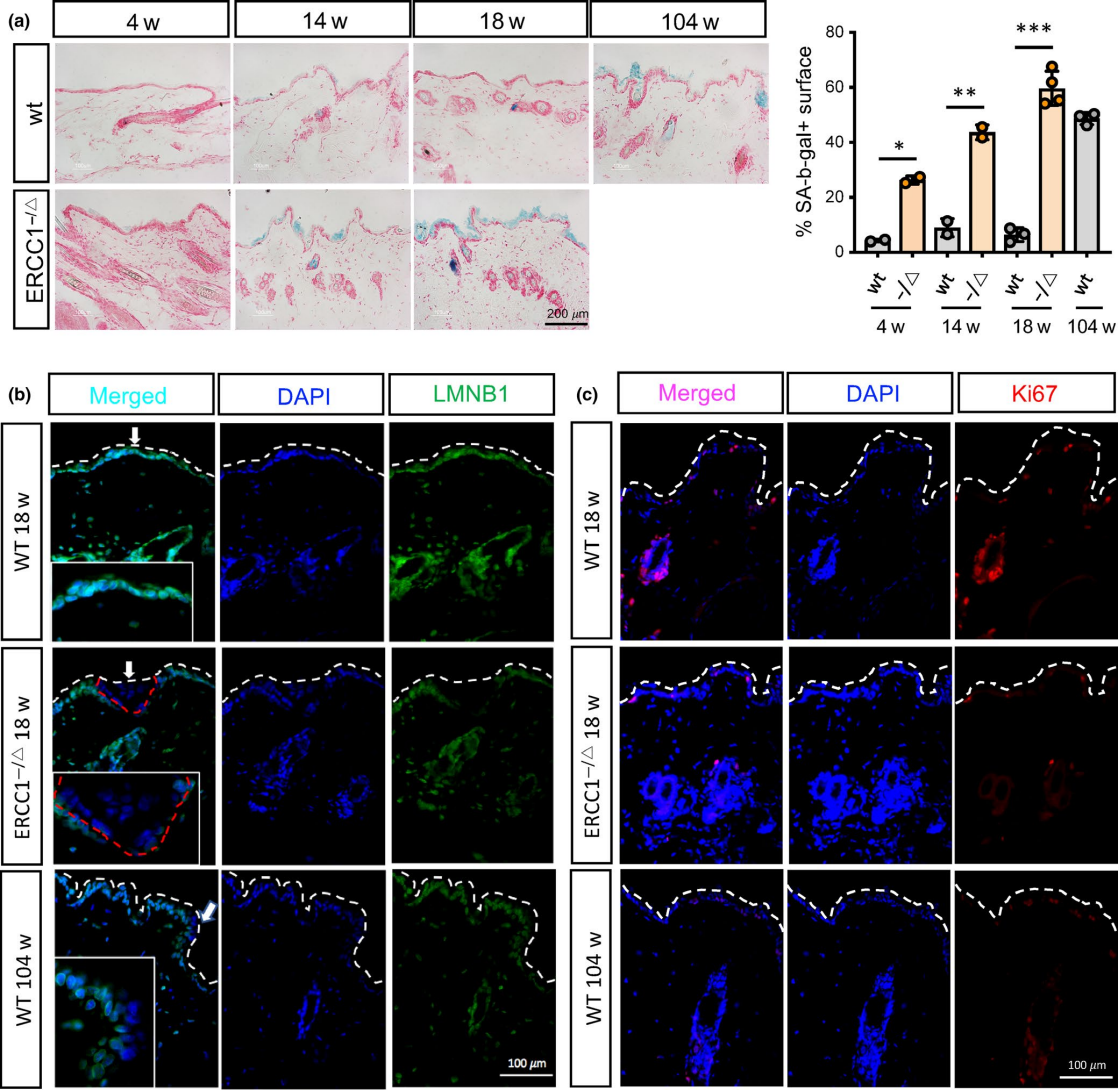
Accumulation of senescent cells in the skin of *Ercc1* ^−/Δ^ mice. (a) *Ercc1* ^−/Δ^ and wild‐type (wt) mouse skin at the indicated ages were stained for senescence‐associated β‐galactosidase (SA‐β‐gal, blue) activity and counterstained with nuclear fast red (red). Representative images (left panel) and quantification (right panel) are shown. *N* = 2–4 animals per condition. Each dot in the graph corresponds to skin from a different animal. Data are mean ± *SEM*, two‐tailed Welch's adjusted *t*‐test. (b, c) Representative images of immunofluorescence staining for lamin B1 (LMNB1) (b) and Ki67 (c) performed on *Ercc1* ^−/Δ^ and wt skin tissue from mice of the indicated genotype and age (w = weeks). DAPI was used for counterstaining. Representative images are shown, and *n* = 3 tissues were evaluated per condition. *p*‐value: *p < .05, **p < .01, ***p < .001

To examine the accumulation of cellular senescence in progressively aged *Ercc1^−/Δ^* mice *in vivo*, we compared skin tissues of *Ercc1^−/Δ^* animals with age‐matched littermates and older wild‐type (wt) control mice. SA‐β‐gal staining showed that the presence of senescent cells in *Ercc1^−/Δ^* skin increased progressively from 4 to 18 weeks of age and was always substantially higher than in skin from similarly aged (4–18 weeks) control wt mice (Figure 1a). Interestingly, skin samples from more substantially aged (104 weeks old) wt mice showed a level of senescent cells comparable to the level observed in 18‐week‐old *Ercc1^−/Δ^* mice. Histological examination of the skin from 18‐week‐old skin of *Ercc1^−/Δ^* ^^mutants showed typical signs of aging such as hyperplasia of the epidermis and atrophy of the dermis (Figure 1a), possibly due to loss and degeneration of the elastic fiber network.

Senescent cells undergo nuclear reorganization, accompanied by a reduction in lamin B1 (LMNB1) mRNA and protein (Freund, Laberge, Demaria, & Campisi, 2012). Immunofluorescence staining of 18‐week‐old *Ercc1^−/Δ^* mouse skin showed a marked loss of nuclear LMNB1 in comparison to age‐matched wt skin, where LMNB1 expression was clearly detectable (Figure 1b and Figure S1A). Similar to the skin of 18‐week‐old *Ercc1^−/Δ^* mice, the skin of 104‐week‐old wt mice displayed a loss of nuclear LMNB1 protein. Staining for the proliferation marker Ki67 showed a significant reduction in the skin of 18‐week‐old *Ercc1^−/Δ^* mice compared to age‐matched wt skin, but similar to aged wt skin (Figure 1c).

**Figure 2:**
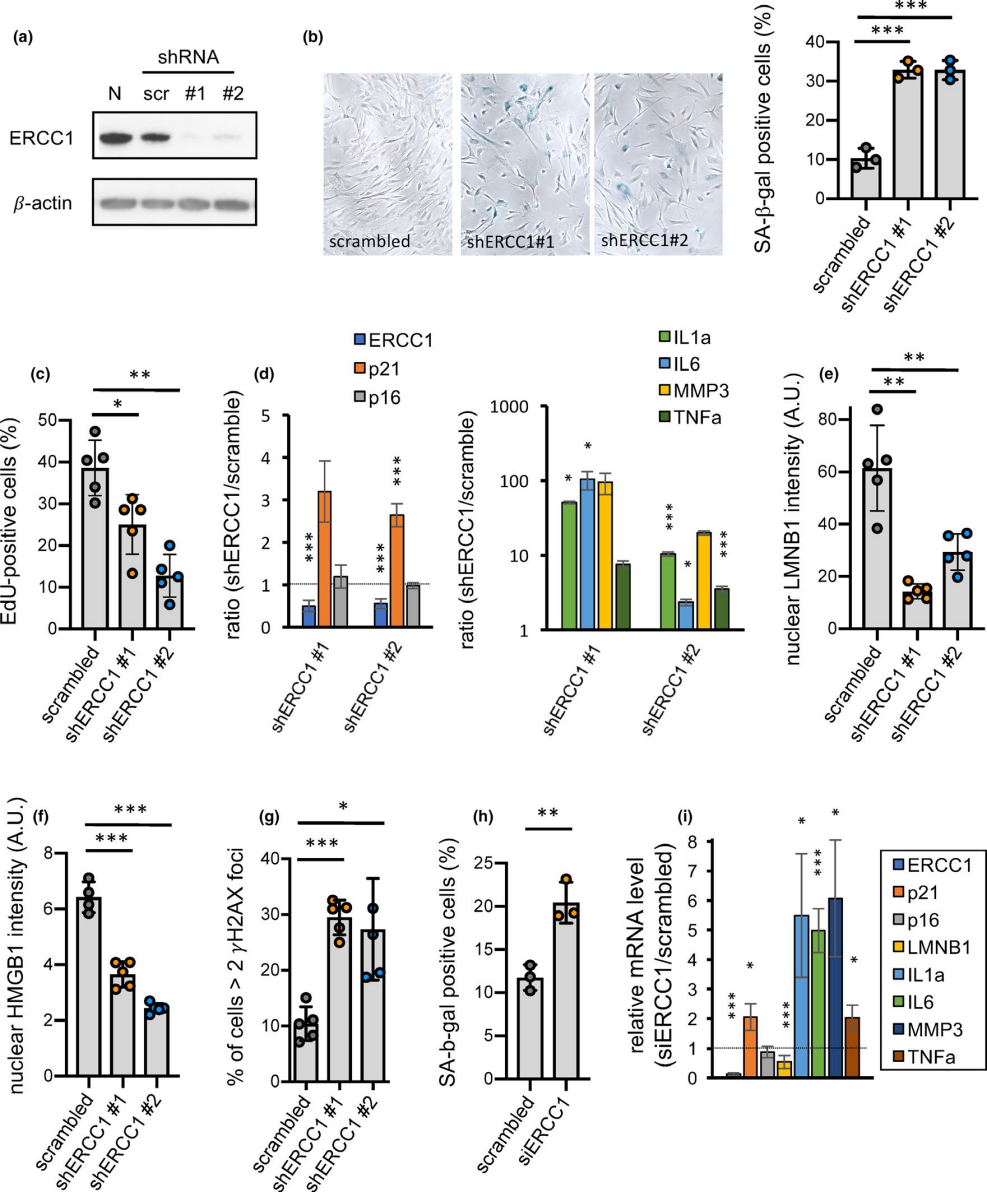
Increased cellular senescence in ERCC1‐deficient human fibroblasts. (a) Human HCA2 skin fibroblasts infected with lentiviruses expressing a scrambled shRNA control (scr) or expressing an shRNA against ERCC1 (shERCC1 #1 and shERCC1 #2) were analyzed by western blotting for ERCC1 protein levels 7 days after infection (β‐actin was used for normalization). N corresponds to noninfected cells. (b) SA‐β‐gal staining was performed on the three populations of human fibroblasts described in A (left panel) and quantification of the percent SA‐β‐gal‐positive cells is shown (right panel). A representative experiment from three independent experiments is shown, *n* = 3, mean ± *SEM*, two‐tailed Welch's adjusted *t*‐test. (c) EdU staining was performed on the three populations, and quantification of the percent EdU‐positive cells is shown. A representative experiment from three independent experiments is shown, *n* = 5, mean ± *SEM*, two‐tailed Welch's adjusted *t*‐test. (d) RNA was extracted from scrambled control, shERCC1 #1 and #2 cell populations, and analyzed by qPCR for mRNA levels of ERCC1 and several senescence markers (p21 and p16 (left panel); IL1α, IL6, MMP3 and TNFα (right panel)). β‐actin was used for normalization. *n* = 3, two‐tailed Welch's adjusted *t*‐test. (e–g) Intensities of nuclear LMNB1 (e) and nuclear HMGB1 (f), and percent cells with at least two γH2AX foci (g), were determined in the three cell populations by immunofluorescence. A representative experiment from 2 independent experiments is shown, *n* = 5 (*n* = 4 only for scrambled in (f)), mean ± *SEM*, two‐tailed Welch's adjusted *t*‐test. (h) SA‐β‐gal staining was performed on human fibroblasts transfected with either a scrambled siRNA control or siRNA against ERCC1 (siERCC1); the percent SA‐β‐gal‐positive cells is shown. *n* = 3, mean ± *SEM*, two‐tailed Welch's adjusted *t*‐test. (i) RNA was extracted from scrambled control and siERCC1 cell populations, and analyzed by qPCR for mRNA levels of ERCC1, and various senescence and SASP markers. β‐actin was used for normalization. *n* = 2, two‐tailed Welch's adjusted *t*‐test. *p*‐value: *p < .05, **p < .01, ***p < .001

To determine whether there is a direct role for ERCC1 deficiency in inducing senescence, we used lentiviruses and two primary human fibroblast strains, HCA2 and IMR‐90, to express two independent short hairpin RNAs (shRNAs) against ERCC1 (shERCC1 #1 and #2). Concomitant with a decrease in ERCC1 protein levels by both shRNAs, there was a significant increase in the number of SA‐β‐gal‐positive cells and a decline in the number of proliferating (EdU positive) cells 7 days after infection (Figure 2a–c). In addition, there was an increase in mRNA encoding the senescence marker p21, but not p16INK4a, 7 days after infection (Figure 2d, left panel; Figure S1B, left panel). However, p16INK4a mRNA increased at later time points, that is, 14 and 21 days after infection (Figure S1C). Further, the expression (mRNA level) of SASP components, such as cytokines (IL1α, IL6, TNFα) and matrix metalloproteinase (MMP3), increased significantly upon ERCC1 depletion (Figure 2d, right panel; Figure S1B, right panel). Immunofluorescence staining confirmed that ERCC1 depletion resulted in a loss of LMNB1 expression (Figure 2e and Figure S1D), loss of nuclear HMGB1 (Figure 2f and Figure S1E), and an increase in γH2AX foci (Figure 2g and Figure S1F), all of which can also serve as senescence markers (Davalos et al., 2013; Rodier et al., 2009). ERCC1 depletion using siRNA further validated the increased number of SA‐β‐gal‐positive cells, as well as changes in expression of the senescence markers measured after shRNA depletion (Figure 2h‐I).

### 2.2 Cellular senescence induced by ERCC1 deficiency is p53‐dependent

**Figure 3:**
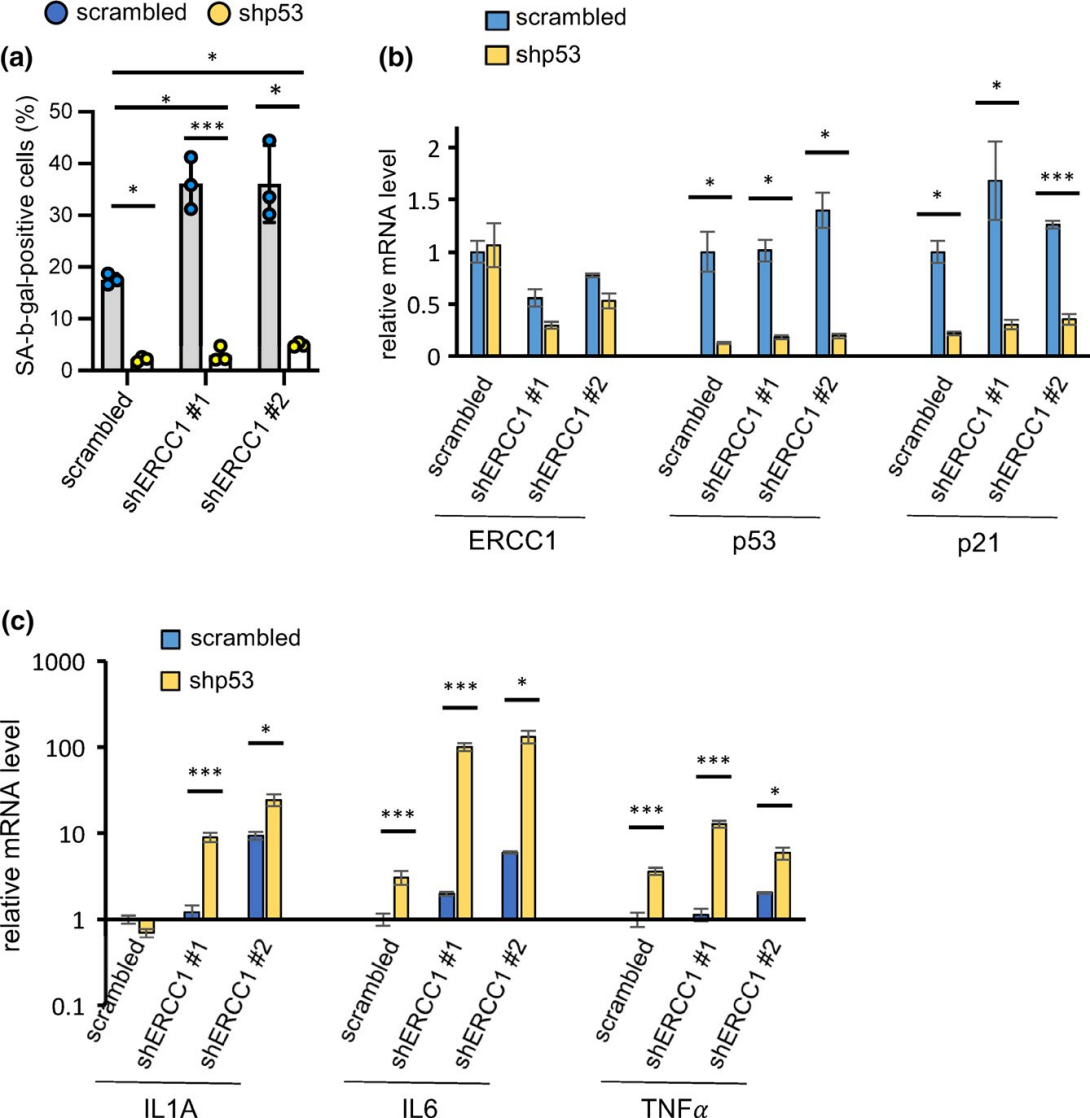
Cellular senescence in ERCC1‐deficient human fibroblasts is p53‐dependent. (a) Quantification of SA‐β‐gal staining is shown for human HCA2 fibroblasts in which knockdown of ERCC1 and/or p53, individually or in combination, was performed using lentivirus‐mediated shRNAs. *n* = 3, mean ± *SEM*, two‐tailed Welch's adjusted *t*‐test. (b, c) RNA was extracted from human fibroblasts infected with shRNAs against ERCC1 and p53, and analyzed by qPCR for several senescence and SASP markers. β‐actin was used for normalization. *n* = 2, two‐tailed Welch's adjusted *t*‐test. *p*‐value: *p < .05, **p < .01, ***p < .001

To determine whether cellular senescence induced by ERCC1 deficiency is p53‐dependent, we used lentivirus‐mediated shRNA depletion of p53 in fibroblasts in which ERCC1 was subsequently depleted. The increased number of SA‐β‐gal‐positive cells normally caused by ERCC1 depletion was substantially attenuated when p53 was depleted as a first step (Figure 3a). Likewise, the downregulation of p53 in ERCC1‐depleted cells dampened the increased mRNA levels of p21, a downstream target of p53, without affecting *ERCC1* mRNA levels (Figure 3b). However, the expression of SASP components (IL1α, IL6, and TNFα mRNAs) was enhanced upon double knockdown of p53/ERCC1 compared to a single knockdown of ERCC1 (Figure 3c), consistent with the negative regulation of the SASP by p53, as reported (Coppe et al., 2008; Rodier et al., 2009). These results indicate that the senescence induced by ERCC1 deficiency is p53‐dependent and confirm that p53 restrains SASP factor expression.

### 2.3 TNFα secreted by senescent cells promotes apoptosis

**Figure 4:**
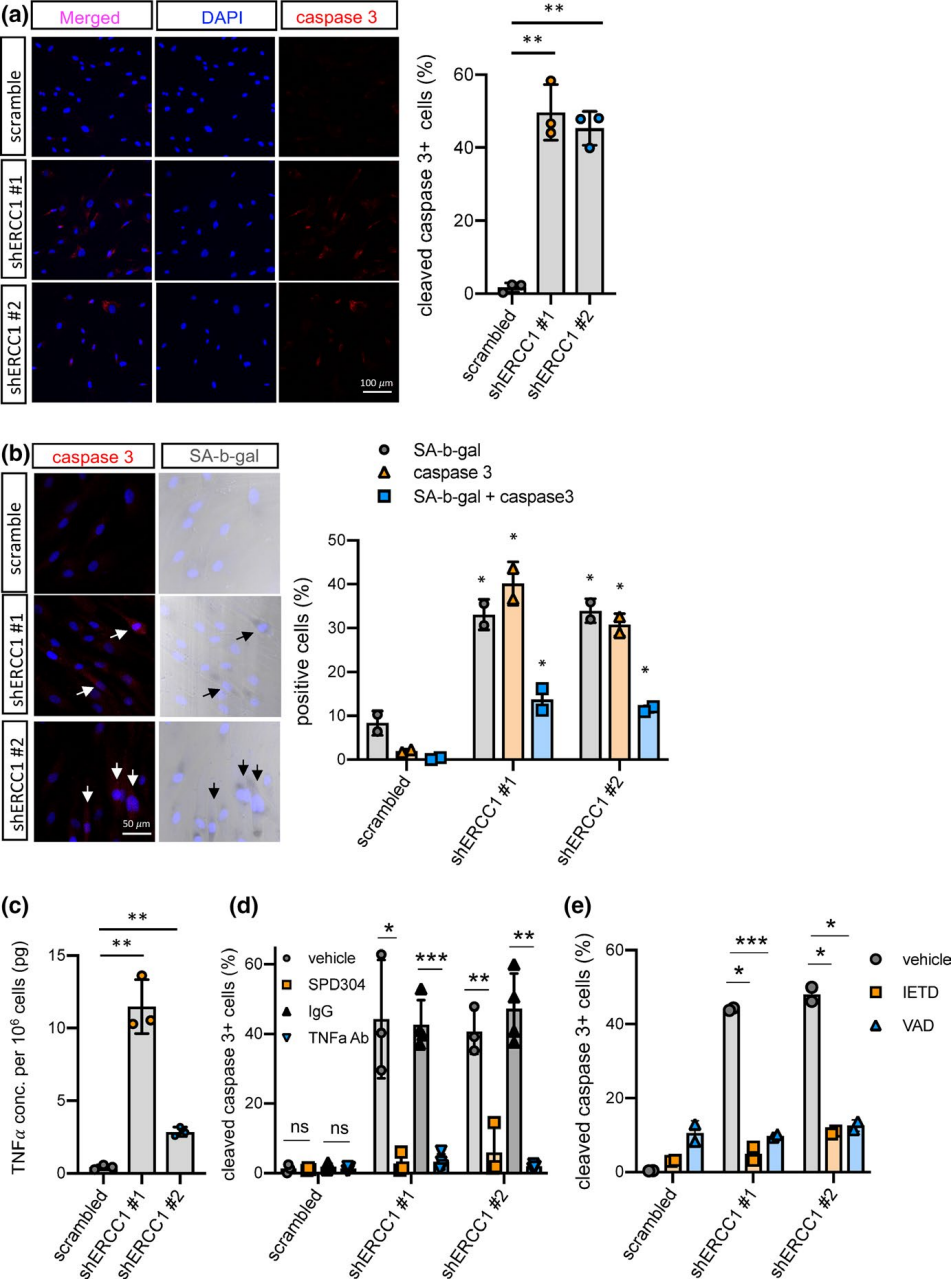
Apoptosis in ERCC1‐deficient human fibroblasts. (a) Representative images (left panel) and quantification (right panel) of immunofluorescence staining for cleaved caspase 3 were obtained using human HCA2 fibroblasts infected with shRNAs against ERCC1 or scrambled shRNA. *n* = 3, at least 5 fields and more than 250 cells counted per condition, mean ± *SEM*, two‐tailed one sample *t*‐test. (b) Representative images (left panel) and quantification (right panel) of SA‐β‐gal staining followed by immunofluorescence staining for cleaved caspase 3 were obtained in human HCA2 fibroblasts infected with shRNAs against ERCC1 or scrambled control. *n* = 2, at least 5 fields and more than 250 cells counted per condition, mean ± *SEM*. (c) TNFα ELISA was performed on conditioned media obtained from human HCA2 fibroblasts infected with shRNAs against ERCC1 or scrambled control. (d–e) Immunofluorescence staining for cleaved caspase 3 in ERCC1‐deficient human fibroblasts treated with 1 μM of the TNFα small molecule inhibitor SPD304 or 100 ng/ml of TNFα blocking antibody (TNFα Ab) (d), as well as 50 μM caspase 8 inhibitor Z‐IETD‐FMK (IETD) or 50 μM caspase 8 inhibitor Z‐VAD‐FMK (VAD) (e), together with vehicle and rabbit IgG controls. *n* = 2 independent experiments. At least 5 fields and more than 200 cells were counted per condition, mean ± *SEM*, two‐tailed Welch's adjusted *t*‐test. *p*‐value: *p < .05, **p < .01, ***p < .001

Our findings suggested that senescent cells might play a causal role in the age‐related phenotypes of *Ercc1* ^−/Δ^ mice. To test this possibility, we bred *Ercc1* ^−/Δ^ mice with p16‐3MR mice and senescent cells were eliminated by treatment with ganciclovir. Eliminating senescent cells neither rescued obvious aging phenotypes nor extended lifespan (Figure S2A), suggesting that other mechanisms drive the phenotypes resulting from Ercc1 deficiency. Indeed, we found that ERCC1 deficiency in human fibroblasts not only induced cellular senescence, but also apoptosis. Immunofluorescence staining for cleaved caspase 3 and TUNEL assays indicated that apoptosis was significantly increased in ERCC1‐deficient fibroblasts (Figure 4a and Figure S2B,C). Senescent cells are reported to be resistant to apoptosis due to increased expression of anti‐apoptotic proteins such as Bcl‐2 (Childs et al., 2014; Yosef et al., 2016). However, upon ERCC1 depletion, these two distinct cellular processes appeared to coexist. To understand how these two processes were related, we performed SA‐β‐gal staining followed by immunofluorescence staining for cleaved caspase 3 (Figure 4b). About 10% of the cells co‐expressed SA‐β‐gal and cleaved caspase 3, suggesting that senescence and apoptosis were concomitant in a fraction of ERCC1‐deficient fibroblasts. However, 20%–25% of ERCC1‐deficient fibroblasts that were not SA‐β‐gal positive also underwent apoptosis. These findings raise the possibility that a paracrine factor produced by senescent ERCC1‐deficient fibroblasts might act on nonsenescent neighboring cells.

TNFα is a SASP factor and known regulator of apoptosis (Jiang, Woronicz, Liu, & Goeddel, 1999). TNFα levels were shown to be significantly higher in sera and various tissues, including fat and endothelium, in *Ercc1* ^−^ *^/Δ^* mice compared to age‐matched wt mice (Chen et al., 2013; Karakasilioti et al., 2013; Roh et al., 2013). Consistent with mRNA levels (Figure 2d, right panel), TNFα secretion by ERCC1‐deficient fibroblasts was significantly higher than control fibroblasts (Figure 4c). TNFα was shown to impede the repair of certain DNA lesions, such as cyclobutane pyrimidine dimers, and increase apoptosis in NER‐incompetent cells via replication stress (Faurschou, Gniadecki, Calay, & Wulf, 2008). We therefore hypothesized that ERCC1‐deficient cells would be susceptible to apoptosis owing to TNFα secretion by senescent cells. To test this hypothesis, we disabled TNFα activity in conditioned media from ERCC1‐deficient cells using a blocking antibody, or blocked TNFα signaling in ERCC1‐deficient cells using a small molecule inhibitor (SPD304) (He et al., 2005). This small molecule inhibitor interferes with protein–protein interactions mediated by glycine 122 residues in the trimeric TNFα receptor type 1 (TNFR1), thereby dissociating the trimer and inhibiting TNFR1 activity. The number of cleaved caspase 3‐positive cells was significantly diminished by treatment with either the TNFα‐blocking antibody or SPD304, as well as an inhibitor of caspase 8, Z‐IETD‐FMK, or a pan‐caspase inhibitor, Z‐VAD‐FMK (Figure 4d‐e). These results indicate that apoptosis induced upon ERCC1 depletion is mainly driven by a TNFα‐dependent extrinsic apoptosis pathway through TNFR1 and that cells rendered NER‐deficient cells by ERCC1 depletion become susceptible to TNFα‐dependent apoptosis by cell autonomous and nonautonomous mechanisms.

**Figure 5:**
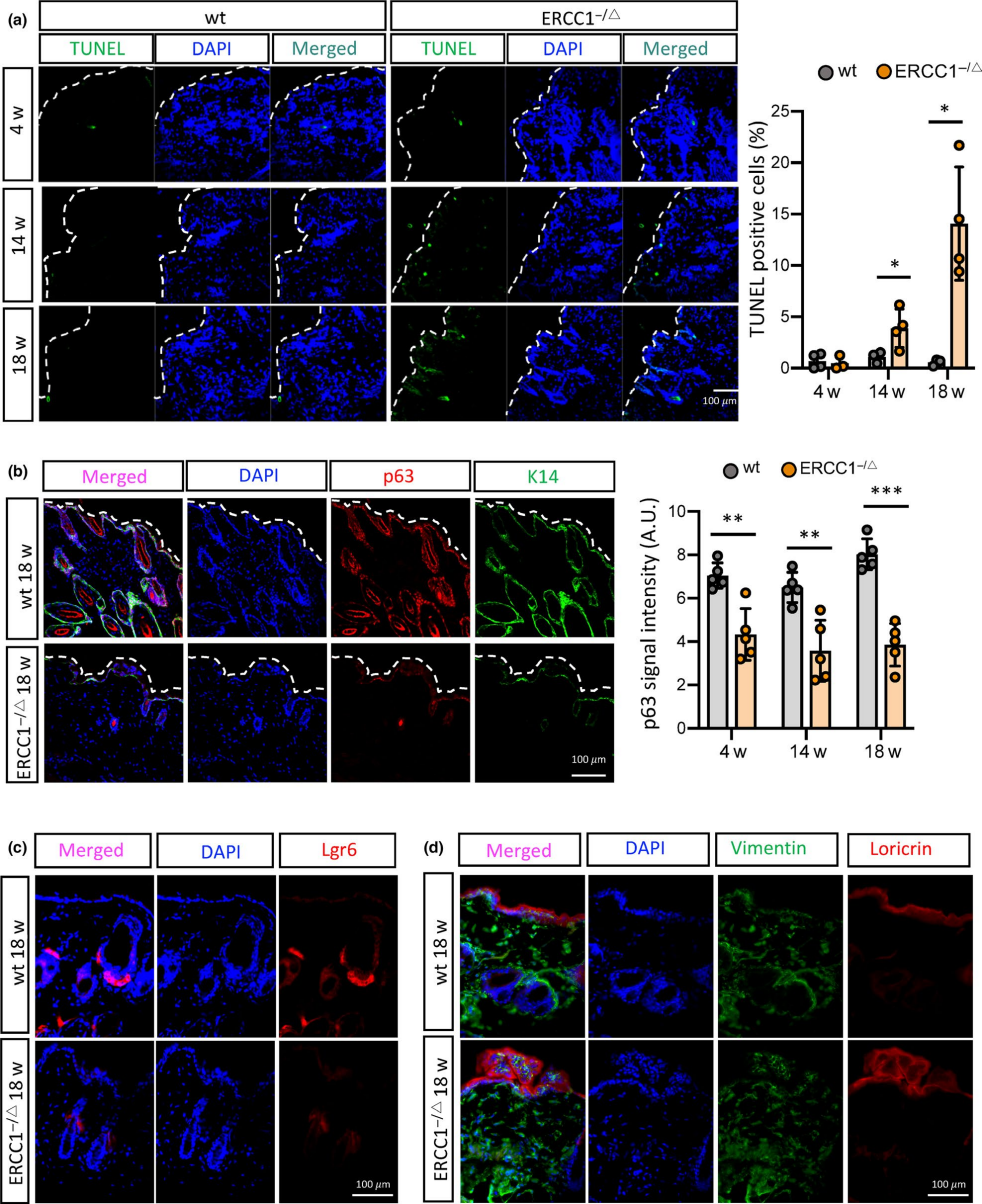
Accumulation of apoptotic cells and depletion of stem cells in the skin of *Ercc1* ^−/Δ^ mice. (a) TUNEL assays in skin of *Ercc1* ^−/Δ^ and wt mice at the indicated ages are shown (left panel); quantification of the percent TUNEL‐positive cells was performed using image J software (right panel). *n* = 3–4 animals per condition, mean ± *SEM*, two‐tailed one sample *t*‐test. (b) Representative images (left panel) of immunofluorescence staining for p63 and K14 were obtained in skin of *Ercc1* ^−/Δ^ and wt mice at 18 weeks of age. Quantification of p63 signal intensity (right panel) was performed on K14‐positive cells. *n* = 5 animals per condition, at least 5 fields and 250 cells counted per condition, mean ± *SEM*, two‐tailed one sample *t*‐test. (c, d) Representative images of immunofluorescence staining for Lgr6 (c), and loricrin together with vimentin (d), were obtained in skin of *Ercc1* ^−/Δ^ and wt mice at 18 weeks of age. *n* = 3 animals per condition, at least 5 fields and 250 cells counted per condition, mean ± *SEM*, two‐tailed Welch's adjusted *t*‐test. *p*‐value: *p < .05, **p < .01

We validated the increase in apoptotic cells *in vivo* using skin from *Ercc1^−/Δ^* as well as age‐matched control mice. TUNEL‐positive cells were clearly evident in epithelium and hair follicles of 14‐week‐old *Ercc1^−/Δ^* skin and were even more abundant by the age of 18 weeks, whereas skin from age‐matched wt mice had very low numbers of TUNEL‐positive cells (Figure 5a). We attempted to perform TUNEL assays along with staining for cleaved caspase 3. However, this experiment was technically challenging. DNase I treatment, a critical step for the TUNEL assay, generated a high background signal for caspase 3 staining, which made interpretation difficult.

Concomitantly, expression of the epithelial stem cell marker (p63), basal stem cell marker (K14), and hair follicle stem cell marker (Lgr6) was significantly decreased in the skin of *Ercc1^−/Δ^* ^^mice (at 4, 14, and 18 weeks of age) compared to age‐matched wt mice (Figure 5b,c). In addition, localization of the terminal differentiation marker, loricrin, was abnormal in the skin of *Ercc1^−/Δ^* ^^mice at 18 weeks of age, indicating a disruption in normal skin structure, possibly due to depletion of stem cell populations (Figure 5d). These data indicate that ERCC1 deficiency in the mouse skin results in cellular senescence followed by apoptosis and suggest that stem cell depletion, likely through apoptosis, may be a major cause of the premature skin aging in *Ercc1^−/Δ^* mice.

## 3 DISCUSSION

Cellular senescence is a cell fate in which cells irreversibly arrest proliferation, in part as a defense mechanism against damage and neoplastic transformation. Emerging evidence indicates that senescent cells accumulate with age in most, if not all, mammalian tissues and organs, including the skin. Senescent cells can alter the tissue microenvironment by secreting SASP factors, which can also create a pro‐inflammatory milieu. Consequently, the SASP can not only promote cancer, but also many chronic and degenerative pathologies associated with aging, ranging from neurodegenerative diseases to diabetes (Campisi, 2013; Palmer et al., 2015). Therefore, senescent cells can be beneficial or deleterious, depending on the context and age of the organism. Likewise, apoptosis, another protective mechanism against stress and cancer, is dysregulated during aging, which can contribute to the deterioration of tissue and organ function (Childs et al., 2014).

Senescent cells are generally resistant to apoptosis, often due to increased expression of anti‐apoptotic proteins belonging to the BCL family. A new class of small molecules, termed senolytics, have now been developed that target these proteins to selectively alter the fate of senescent cells, causing them to die by apoptosis (Chang et al., 2016; Yosef et al., 2016). Most senescent cells resist apoptotic death, although senescent endothelial cells have been reported to undergo a cell fate transition to apoptosis by reducing anti‐apoptotic protein levels or increasing pro‐apoptotic protein levels (Li et al., 2005; Matsushita et al., 2001; Zhang et al., 2002). Indeed, we determined that Bcl2 mRNA levels were reduced in ERCC1‐depleted HCA2 fibroblasts (data not shown). Here, we show that ERCC1 depletion in human fibroblasts induces cellular senescence and TNFα, one of the SASP factors secreted by senescent cells, which promotes apoptosis in neighboring cells and in senescent cells themselves due to their defect in DNA repair (Figure S2D). However, we cannot rule out the possibility that the results depend on the stage of senescent cells.

ERCC1 depletion in several cell types caused micronuclei formation, which was linked to chromosome mis‐segregation (Melton et al., 1998; Niedernhofer et al., 2004). Using DAPI staining, we also observed micronuclei in ERCC1‐deficient fibroblasts. Although we have not tested this hypothesis directly, it is conceivable that the formation of micronuclei and possibly cytosolic DNA triggers the cGAS‐STING pathway, thereby activating NF‐κB and elevating type I IFN signaling, both of which were recently shown to occur in senescent cells (De Cecco et al., 2019). Indeed, Tilstra et al. reported that NF‐κB activation was significantly enhanced in multiple tissues from *Ercc1* ^–/Δ^ mice, including kidney, liver, pancreas, spleen and muscle (Tilstra et al., 2012). In addition, it was reported that IFN‐γ activates the transcription of TNFα (Vila‐del Sol, Punzon, & Fresno, 2008)*.* Thus, the DNA repair defects in *Ercc1*‐deficient cells could possibly result from activation of the cGAS‐STING‐type I IFN pathway, subsequent NF‐κB activation, followed by TNFα‐induced apoptosis.

ERCC1 participates in several, related DNA repair processes, including NER, TCR and interstrand crosslinking repair (Dolle et al., 2011; Weeda et al., 1997). *Ercc1^−/Δ^* mice are a model for human Cockayne and COF syndromes, which are rare progeroid syndromes with extremely short lifespans (Gregg et al., 2011; Vermeij, Dollé, et al., 2016). Increases in both cellular senescence and apoptosis in *Ercc1^−/Δ^* mice have been reported (Niedernhofer et al., 2006; Robinson et al., 2018; Takayama et al., 2014; Tilstra et al., 2012; Weeda et al., 1997). These two processes are partly interconnected by common molecular pathways (Childs et al., 2014), but how they interact is not clear. Our surprising finding that both processes are induced, presumably sequentially, by ERCC1 depletion identifies a potential mechanism – namely that apoptosis is caused by a SASP factor, TNFα, secreted by senescent cells. Yet, this TNFα secretion promotes apoptosis only under the circumstances where DNA repair is compromised. Indeed, although TNFα can be a strong inducer of apoptosis, it cannot trigger apoptosis in normal cells that are capable of rapid DNA damage repair (Sedger & McDermott, 2014). However, ERCC1‐depleted cells that are deficient in NER become susceptible to TNFα‐driven apoptosis. It has been shown that TNFα increases the proportion of cells entering S phase and thus causes replication stress, which can result in apoptosis in NER‐incompetent premalignant cells (Faurschou et al., 2008). Here, it is also possible that TNFα secreted by ERCC1‐depleted senescent cells could trigger entry into S phase, and thus induce apoptosis in DNA repair‐deficient neighboring cells. In addition, TNFα secretion could induce apoptosis in a cell autonomous manner.

Recent findings show that caloric restriction can be an effective therapeutic intervention in *Ercc1^−/Δ^* mice by reducing the burden of DNA damage (Vermeij, Dollé, et al., 2016). Restricting calories has also been shown to reduce serum levels of TNFα (Phillips & Leeuwenburgh, 2005), suggesting that caloric restriction might significantly reduce the upregulation of TNFα in *Ercc1^−/Δ^* mice (Chen et al., 2013; Karakasilioti et al., 2013; Roh et al., 2013). This dampening could in turn diminish TNFα‐induced apoptosis, which could delay the accelerated aging phenotypes and possibly explain the extended median and maximal lifespans observed in calorie restricted *Ercc1^−/Δ^* mice (Vermeij, Dollé, et al., 2016).

Anti‐TNFα therapy is approved to treat many inflammatory diseases, including rheumatoid arthritis, psoriasis and Crohn's disease (Sedger & McDermott, 2014). Blocking TNFα signaling effectively prevents the induction of inflammation mediators such as IL‐6 (Gupta, 2002). Here, we suggest a novel anti‐TNFα therapeutic approach to prevent apoptosis by TNFα signaling. Anti‐TNFα therapy could present an alternative intervention in *Ercc1^−/Δ^* mice to counteract aging phenotypes, such as those in the skin, although anti‐TNFα therapy would reduce apoptosis without preventing DNA damage.

In 4‐week‐old *Ercc1^−/Δ^* mice, which was the age at which we began eliminating senescent cells, these cells already accumulated and secreted TNFα that could trigger apoptosis in neighboring cells. We speculate that this is why we failed to rescue any aging phenotype and could not detect any effect on health or lifespan. However, we cannot rule out the possibility that clearance of p16‐positive senescent cells did not affect ERCC1 depletion‐induced phenotypes because senescence in these animals is primarily driven by p21. By 14 weeks of age, apoptotic cells were clearly apparent and stem cell pools were significantly depleted. Therefore, as a more compelling and long‐term therapy, we suggest a combination of eliminating senescent cells in a timely fashion and replenishing stem cells might reduce the DNA damage response (Ocampo et al., 2016), and delay or rejuvenate the aging phenotypes in *Ercc1^−/Δ^* mice.

In humans, ERCC1 mutations at residues 158 and 231, yielding a premature Q158X termination mutation, and a F231L mutation in the XPF‐interacting domain have been reported to cause Cockayne and COFS syndromes (Gregg et al., 2011; Jaspers et al., 2007). Patients with Cockayne and COFS syndromes develop postnatal brain growth failure and degeneration of multiple tissues, resulting in cachexia, dementia and premature aging, as well as a shortened lifespan (Wilson et al., 2016) as observed in *Ercc1^−/Δ^* mice. Unfortunately, the current treatment for those patients is limited to symptomatic support (Wilson et al., 2016). Our findings suggest a novel therapeutic intervention for long‐term health benefits in these patients, namely stem cell pool replenishment along with the clearance of senescent cells.

## 4 EXPERIMENTAL PROCEDURES

### 4.1 Cell cultures

HCA2 (from O. Pereira‐Smith) and IMR90 (from American Type Culture Collection) human fibroblasts were cultured in DMEM supplemented with 10% FBS (Invitrogen) and penicillin/streptomycin at 3% oxygen and 10% CO_2_.

### 4.2 Chemicals and treatments

The TNFα‐blocking antibody was from Cell Signaling Technology, the small molecule TNFα inhibitor (SPD304), which interferes with protein–protein interactions mediated by glycine 122 residues in the trimeric TNFα receptor type 1 (TNFR1), thereby dissociating the trimer and inhibiting TNFR1 activity, was from Sigma‐Aldrich, and the caspase 8 (Z‐IETD‐FMK) and pan‐caspase (Z‐VAD‐FMK) inhibitors were from R&D Systems. Human fibroblasts were seeded and, the next day, infected with pLKO.1‐ERCC1 or control lentiviruses, followed by treatment every other day with 100 ng/ml TNFα‐blocking antibody, 1 μM SPD304, 50 μM Z‐IETD‐FMK or 50 μM Z‐VAD‐FMK, together with vehicle controls.

### 4.3 Animals

Animal studies were conducted in compliance with protocols approved by the ethics committee of the National Institutes for Public Health and the Environment (RIVM) in the Netherlands. *Ercc1^+/Δ^* and *Ercc1* *^+/−^* mice (from genetically pure C57BL6J and FVB backgrounds) were crossed to obtain *Ercc1^−/Δ^* offspring with a genetically uniform F1 C57BL6J/FVB hybrid background (Dolle et al., 2011; Weeda et al., 1997). Typical unfavorable characteristics, such as blindness in the FVB background or deafness in the C57BL6J background, do not occur in this hybrid background. Since *Ercc1^−/Δ^* mice are smaller, food was administered within the cages and water bottles with long nozzles were used from ~2 weeks of age. Animals were maintained in a controlled environment (20–22°C; 12 hr light:12 hr dark cycle). Wild‐type F1 littermates at the indicated ages were used as controls. All animals were bred and maintained on AIN93G synthetic pellets (Research Diet Services B.V.; gross energy content 4.9 kcal/g dry mass, digestible energy 3.97 kcal/g). To eliminate senescent cells in *Ercc1^−/Δ^*/p16‐3MR ± mice, mice were intraperitoneally injected with a single daily dose of 25 mg ganciclovir (GCV) per kg every 4th week for 5 consecutive days, starting from 4‐weeks of age (*n* = 8 per gender and treatment). Control mice were treated with vehicle (PBS).

### 4.4 Genetic manipulations and gene expression analysis

Lentiviral vectors (pLKO.1‐puro) encoding shRNAs against ERCC1 were purchased from Dharmacon. pLKO.1‐ERCC1 lentiviruses were packaged in HEK293FT and supernatants were collected after 48 hr. Cells were infected with the titrated lentiviruses, followed by selection in 1 μg/ml puromycin for 3 days. For siRNA experiments, cells were transfected with 50 pmole ERCC1 siRNA (Santa Cruz Biotechnology) using Lipofectamine 2000 (Invitrogen) for 8 hr, as per the manufacturer's recommendation. Cells were collected for analysis 7 days after infection or transfection. 1 μg total RNA was used to prepare cDNA using the High Capacity cDNA Reverse Transcription Kit (Life Technologies), followed by real‐time qPCR using the Roche Universal Probe Library system and a Light Cycler 480 (Roche). All samples were analyzed in triplicate using gene‐specific primers and were normalized to β‐actin (Wiley et al., 2016).

### 4.5 SA‐β‐gal staining and EdU labeling

Cells were fixed and processed for senescence‐associated β‐galactosidase (SA‐β‐gal) staining as per the manufacturer's instruction (Biovision). Skin tissues, frozen in OCT, were cut (7–8 μm sections) and processed for SA‐β‐gal and Fast Red staining, as described (Velarde, Demaria, Melov, & Campisi, 2015; Wiley et al., 2016). A Nikon Eclipse E800 microscope was used for imaging and images were quantified using Image J software.

Cell proliferation was evaluated by incorporation of 5‐ethynyl‐2′‐deoxyuridine (EdU) and the Click‐iT EdU Cell Proliferation Assay Kit (Invitrogen). Briefly, cells were given 10 μM EdU for 24 hr before fixation, permeabilized and incubated with Click‐iT reaction cocktail as per the manufacturer's instructions. A Zeiss LSM780 confocal microscope was used for imaging, and images were quantified using Image J software. >100 cells from 5–7 different fields were quantified per condition, and all experiments were done in duplicate.

### 4.6 Immunofluorescence staining

Human cells and skin cryosections (7–8 μm) were fixed in 4% paraformaldehyde, permeabilized, blocked and immunostained as described (Davalos et al., 2013; Freund et al., 2012; Rodier et al., 2009). Primary antibodies were rabbit anti‐ERCC1 (Santa Cruz #sc‐10785), rabbit anti‐γH2AX (Novus #NB100‐79967), rabbit anti‐HMGB1 (Abcam #ab18256), goat anti‐LMNB1 (Santa Cruz #sc‐6217), rabbit anti‐cleaved caspase 3 (Cell Signaling #9664), rabbit anti‐p63 (GeneTex #GTX102425), chicken anti‐keratin14 (BioLegend #906001), rabbit anti‐Lgr6 (Abcam #ab126747), rabbit anti‐loricrin (Covance #PRB‐145P) and chicken anti‐vimentin (Abcam #ab24525). Secondary antibodies were Alexa Fluor 488 anti‐mouse, Alexa Fluor 488 anti‐goat, Alexa Fluor 488 anti‐chicken and Alexa Fluor 597 anti‐rabbit IgG from Molecular Probe. A Zeiss LSM780 confocal microscope was used for imaging, and images were quantified using Image J software. >200 cells in 5–12 different fields were quantified per condition.

### 4.7 Immunoblotting

Cells were lysed and proteins separated by SDS‐PAGE using 4%–12% Bis‐Tris gels, followed by transfer to PVD membranes, blocking, incubation with primary antibodies overnight at 4°C, incubation with HRP‐conjugated secondary antibodies for 1 hr at room temperature, and detection using enhanced chemiluminescence. Primary antibodies were rabbit anti‐ERCC1 (Santa Cruz #sc‐10785) and mouse anti‐β‐actin (Sigma #A2228). Secondary antibodies were HRP‐conjugated goat anti‐rabbit (Bio‐Rad) and goat anti‐mouse (Bio‐Rad) antibodies.

### 4.8 TUNEL assay

Frozen skin tissues were cut, fixed and processed for enzymatic reaction using the Dead End Fluorometric TUNEL System (Promega) as per the manufacturer's instruction. A Zeiss LSM780 confocal microscope was used for imaging and the images were quantified using Image J software. At least three different fields with >300 cells per field were quantified per condition, and tissues from three different animals were used for the assay.

### 4.9 TNFα ELISA

Cells were infected with lentivirus expressing ERCC1 shRNA as well as a scrambled shRNA, and cultured for 7 days. Conditioned media were collected from cells cultured in 0.2% serum for 24 hr, followed by concentration using Amicon Ultra Centrifugal Filter units (Millipore). The concentrated conditioned media were used to measure TNFα concentrations as per the manufacturer's instruction (TNFα ELISA kit from R&D Systems). Cell number was determined and used for normalization.

### 4.10 Statistics

All data with error bars are presented as mean ± *SEM*, and the individual data points (dots) are presented in the bar graphs (Figures 1a, 2b,c, 2e‐h, 3a, 4a‐e, 5a,b, and Figure S2C). We used Welch's adjusted *t*‐test (also called unequal variances *t*‐test), a modified Student's *t*‐test under the assumption of unequal variances. Most of cell culture experiments were done in triplicate and reproduced at least three times independently.

## CONFLICT OF INTEREST

The authors declare no competing financial interests. JC is a founder of Unity Biotechnology, which develops methods to eliminate senescent cells.

## AUTHOR CONTRIBUTIONS

DEK, MD and JC designed the experiments. DEK, MD, CDW and ARD conducted the experiments. MD, WPV, AG, KV and JH provided and analyzed mouse tissue samples. DEK, PYD, PH and JC analyzed the data. DEK, PYD and JC wrote the manuscript. MD, WPV, PH and JH edited the manuscript.

## Data Availability

All data generated or analyzed during this study are included in this published article (and its supplementary information files).
