# Supplementary figures and images for: Deficiency in the DNA repair protein ERCC1 triggers a link between senescence and apoptosis in human fibroblasts and mouse skin
Source: Aging Cell. 2019 Nov 18;19(3):e13072. doi: 10.1111/acel.13072 (PMC7059167; doi:10.1111/acel.13072)

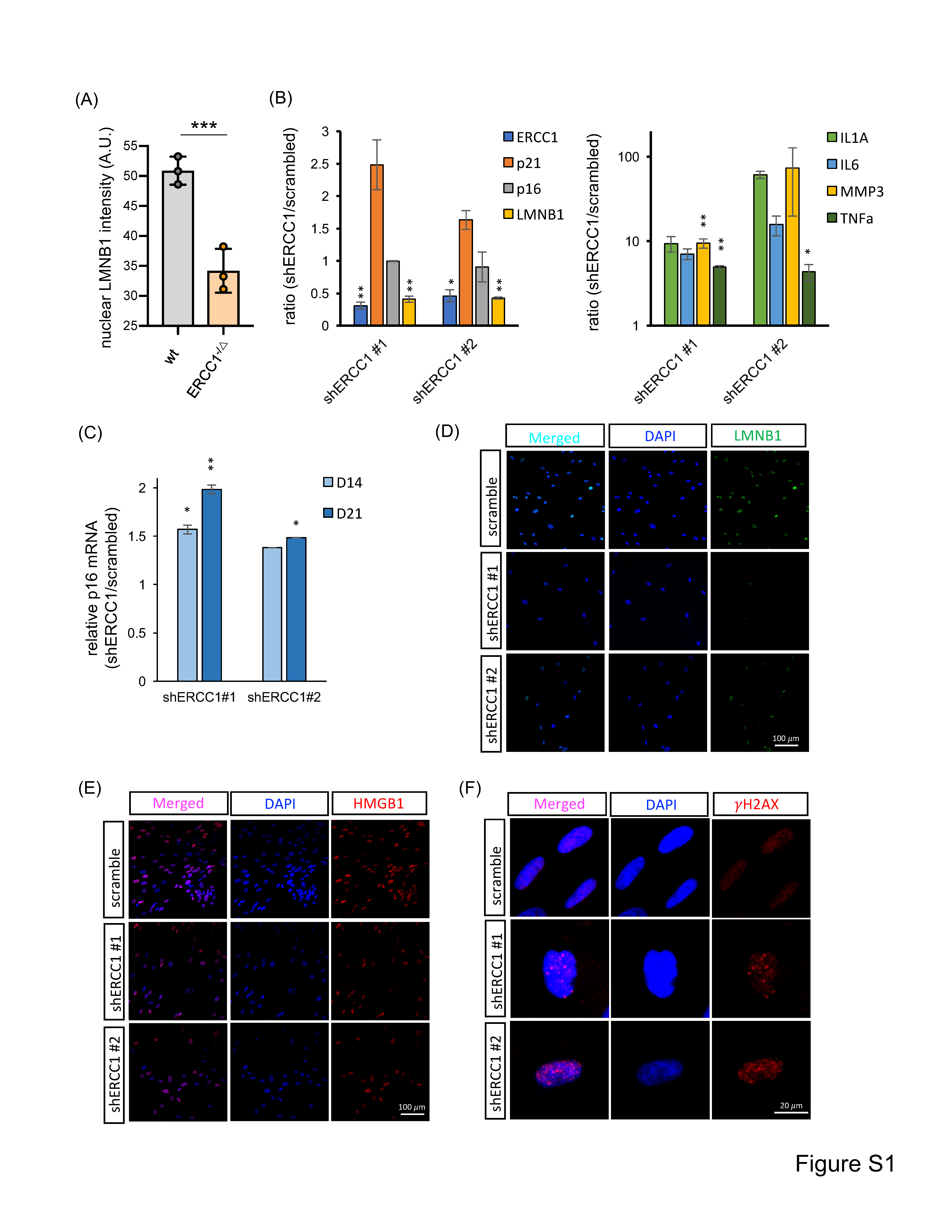

Supplement: Supplementary file 1 [file ACEL-19-e13072-s001.png]

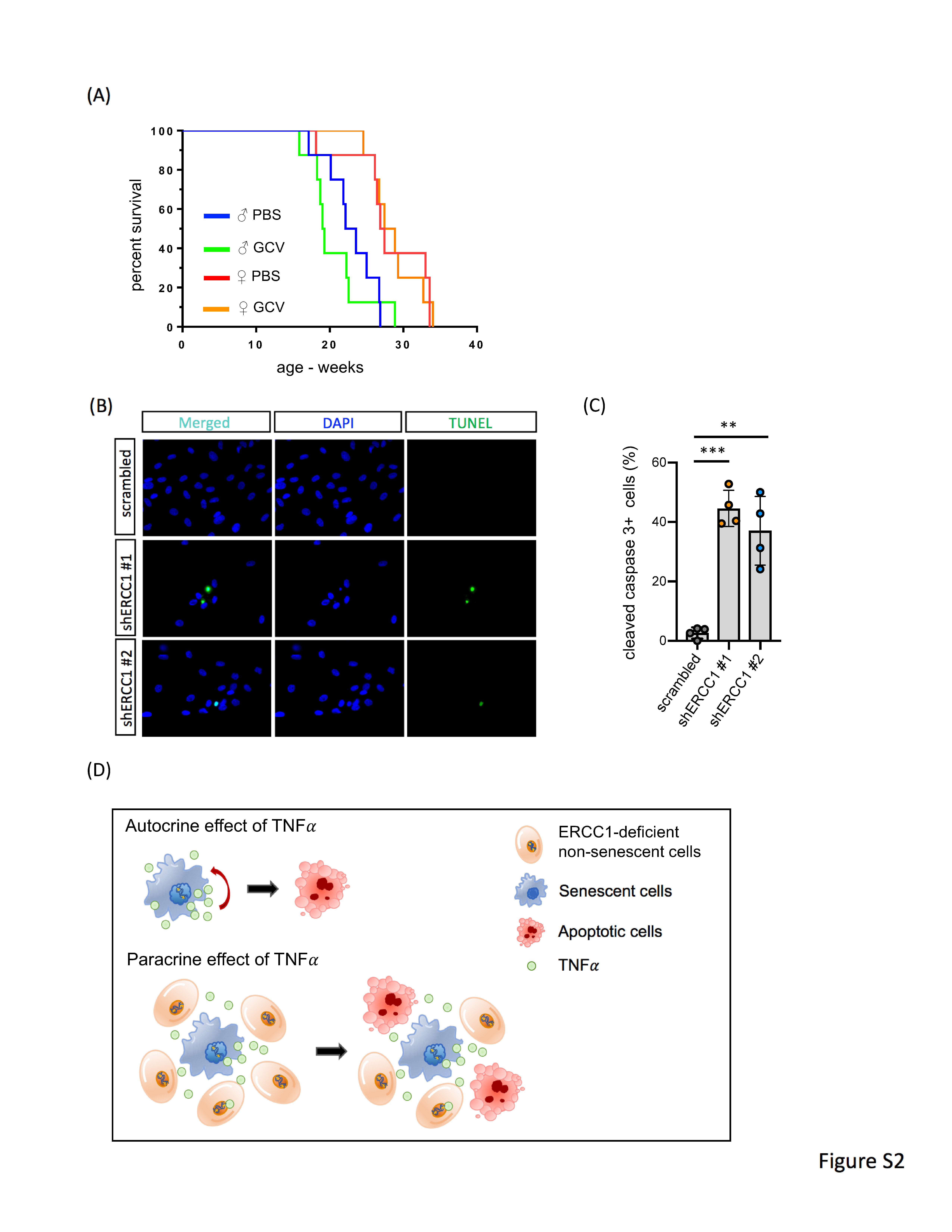

Supplement: Supplementary file 2 [file ACEL-19-e13072-s002.png]
